# Combined integrin α_v_β_3_ and lactoferrin receptor targeted docetaxel liposomes enhance the brain targeting effect and anti-glioma effect

**DOI:** 10.1186/s12951-021-01180-0

**Published:** 2021-12-23

**Authors:** Na Qi, Shangqian Zhang, Xiantai Zhou, Wenjuan Duan, Duan Gao, Jianfang Feng, Aimin Li

**Affiliations:** 1 Cancer Center, Integrated Hospital of Traditional Chinese Medicine, Southern Medical University, Guangzhou, 510315 China; 2 Department of Pharmacy, Guilin Medical University, Guilin, 541004 China; 3 Department of Pharmacy, Guangxi University of Chinese Medicine, Nanning, 530299 China

**Keywords:** Lactoferrin, RGD, Dual-targeting liposomes, Docetaxel, Treatment of glioma

## Abstract

The integrin α_v_β_3_ receptor and Lactoferrin receptor (LfR) are over-expressed in both cerebral microvascular endothelial cells and glioma cells. RGD tripeptide and Lf can specifically bind with integrin α_v_β_3_ receptor and LfR, respectively. In our study, RGD and Lf dual-modified liposomes loaded with docetaxel (DTX) were designed to enhance the brain targeting effect and treatment of glioma. Our in vitro studies have shown that RGD-Lf-LP can significantly enhance the cellular uptake of U87 MG cells and human cerebral microvascular endothelial cells (hCMEC/D3) when compared to RGD modified liposomes (RGD-LP) and Lf modified liposomes (Lf-LP). Free RGD and Lf competitively reduced the cellular uptake of RGD-Lf-LP, in particular, free RGD played a main inhibitory effect on cellular uptake of RGD-Lf-LP in U87 MG cells, yet free Lf played a main inhibitory effect on cellular uptake of RGD-Lf-LP in hCMEC/D3 cells. RGD-Lf-LP can also significantly increase penetration of U87 MG tumor spheroids, and RGD modification plays a dominating role on promoting the penetration of U87 MG tumor spheroids. The results of in vitro BBB model were shown that RGD-Lf-LP-C6 obviously increased the transport of hCMEC/D3 cell monolayers, and Lf modification plays a dominating role on increasing the transport of hCMEC/D3 cell monolayers. In vivo imaging proved that RGD-Lf-LP shows stronger targeting effects for brain orthotopic gliomas than that of RGD-LP and Lf-LP. The result of tissue distribution confirmed that RGD-LF-LP-DTX could significantly increase brain targeting after intravenous injection. Furthermore, RGD-LF-LP-DTX (a dose of 5 mg kg^−1^ DTX) could significantly prolong the survival time of orthotopic glioma-bearing mice. In summary, RGD and LF dual modification are good combination for brain targeting delivery, RGD-Lf-LP-DTX could enhance brain targeting effects, and is thus a promising chemotherapeutic drug delivery system for treatment of glioma.

**Supplementary Information:**

The online version contains supplementary material available at 10.1186/s12951-021-01180-0.

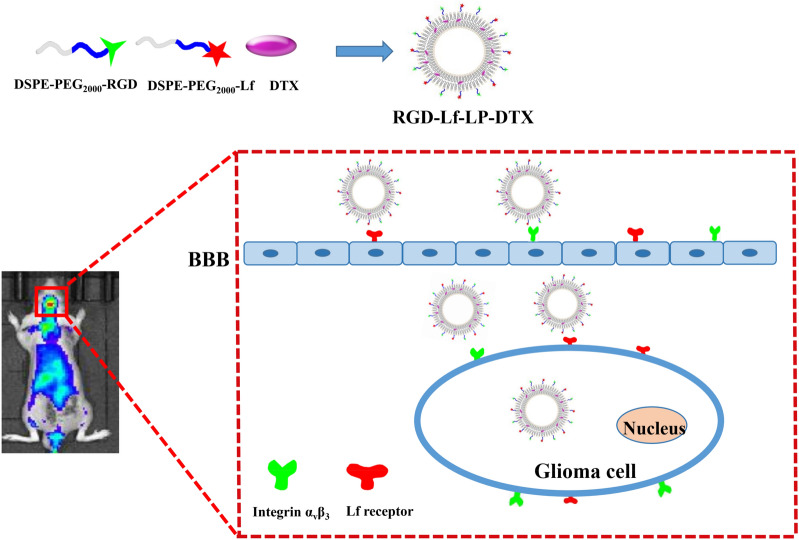

## Introduction

The incidence of glioma is about 5–10 per 100,000 population, and for patients with glioma, the median survival time is only 12–18 months [1, 2]. The standard clinical treatments comprise surgical resection, radiotherapy, and chemotherapy [3]. Surgical resection of glioma is often incomplete, and the blood–brain barrier (BBB) blocks most therapeutic drugs, effectively preventing therapies from reaching the glioma cells, which often leads to glioma recurrence [4, 5]. Providing adequate chemotherapeutic treatment for glioma is still one of the most challenging tasks in oncological clinical practice. The BBB has long been deemed the major obstruction, because it stringently limits the delivery of drugs (and many other substances) from the blood to the brain. Discovering better methods of crossing the BBB is an ongoing and active area of scientific research, and many researchers (both past and present) are working on the development of glioma targeting delivery systems that more effectively cross the BBB [5].

Integrin α_v_β_3_ is an important receptor, known for its role in cancer therapy and the overexpression of cerebral microvascular endothelial cells and glioma cells [4]. The RGD tripeptide can specifically bind to integrin α_v_β_3_, and its transmembrane mechanism may be related to endocytosis mediated by the integrin α_v_β_3_ receptor [2]. Some studies have reported that the RGD targeting α_v_β_3_ integrin receptor has great potential for both the diagnosis and treatment of glioma [3, 4].

Likewise, Lactoferrin (Lf, MW ~ 80 kDa) is a globular glycoprotein. Lf belongs to the family of transferrin (Tf), which specifically binds to the Lf receptors (LfR) in the BBB [6]. Extensive histological studies have showed that LfR is expressed on the cerebral microvascular endothelial cells [7]. In fact, LfR is also expressed on glioma cells [8]. Therefore, conjugated Lf can cross the BBB and target glioma through LfR-mediated endocytosis [9].

In recent, liposomes have been proven to be most useful anticancer agent carriers [10]. Long-circulating (PEGylated) liposomes relies on the PEGylated modification containing hydrophilic groups to extend the circulation time and avoid rapid clearance by mononuclear phagocytic system (MPS) [10, 11]. PEGylated liposomes can enhance permeability and retention (EPR) effect. Furthermore, specific ligand modified delivery system could significantly enhance the drug accumulation into tumor cells and improve its therapeutic efficacy.

Recently, dual ligand modified delivery system showed more promising approach to deliver drug to tumor cells for cancer treatment [12–14]. Scientists have built dual targeting delivery systems as a means of by passing the BBB, these systems using two highly effective targeting ligands modification are better than that of a single ligand modification [11, 14].

DTX is an anti-mitotic taxane drug, which can induce G_2_/M phase arrest of tumor cells and inhibit tumor proliferation. DTX has significant inhibitory effects on various tumors, such as gastric, prostate, and breast tumors, and also non-small cell lung cancer [15]. DTX can induce glioma apoptosis and produce significant inhibitory effects [1]. In addition, DTX is considered one of the most promising drugs for treating brain tumors [9]. However, due to the poor water solubility and low bioavailability of DTX (brand name Taxotere®) the drug has serious side effects and poor targeted delivery efficiency. Despite these limitations, a safer and more efficient drug delivery system can improve the patient tolerance and therapeutic potential of DTX.

In view that RGD and LF specifically recognizes integrin αvβ3 receptor and LfR on the surface of cerebral microvascular endothelial cells and glioma cells, respectively. In our study, PEGylated liposomes were used as drug carriers of brain targeting delivery system, we developed RGD and Lf dual modified liposomes loaded with DTX (RGD-Lf-LP-DTX) to achieve better targeting and treatment of glioma by dual modification. The delivery system was characterized by particle size, zeta potential, morphology, encapsulation efficiency, and drug loading. Tissue distribution, in vitro and in vivo targeting and anti-glioma effects of modified liposomes were evaluated [3].

## Materials and methods

### Materials

DTX was purchased from Wuhan Xinxin Jiali Biological Technology Co, Ltd (Wuhan, China). RGD, Lf, and coumarin-6 (C6, purity > 98%) were obtained from Sigma Aldrich (St. Louis, USA). Cholesterol (Chol) was purchased from Shanghai Xingzhi Chemical Factory Co, Ltd (Shanghai, China). Soya phosphatidyl choline (SPC) was obtained from Lipoid GmbH (Ludwigshafen, Germany). The DSPE-PEG2000 (1,2-distearoyl-sn-glycero-3-phosphoethanolamine-N- [methoxy(polyethyleneglycol)-2000]), DSPE-PEG_2000_-maleimide (DSPE-PEG_2000_-Mal), and DSPE-PEG_2000_-RGD were purchased from Ponsure Co, Ltd (Shanghai, China). 2-iminothiohne (Traut’s) was purchased from Thermo Fischer Scientific (Massachusetts, USA). Sephadex G-50, Sepharose CL-4B, Trypsin, 3-(4,5-dimethylthiazol-2-yl)-2,5-diphenyltetrazolium bromide (MTT), and TritonX-100 were purchased from Solarbio Co, Ltd (Beijing, China). Hoechst 33342 and DiR dye were purchased from YEASEN Co, Ltd (Shanghai, China). DMEM medium was obtained from Gibco (California, USA). Fetal bovine serum (FBS) was purchased from GEMINI (California, USA). The high-performance liquid chromatography (HPLC)-grade solvents used for HPLC were purchased from Xilong Chemical Co, Ltd. (Shantou, China). All other chemicals utilized were analytical grade preparations.

### Cells and animals

U87 MG glioma cells and immortalized hCMEC/D3 were purchased from Shanghai Guandao Biological Engineering Co, Ltd (Shanghai, China). All cells were cultured in DMEM medium, supplemented with 10% FBS and 1% antibiotics (penicillin and streptomycin), and all the cells were likewise cultured at 37 ℃, 5% CO_2_. The male BALB/c nude mice (16–20 g) were obtained from the Shanghai Silaike Laboratory Animal Co, Ltd (Hunan China). All our experiments were performed in compliance with guidelines set by the Guilin Medical University Institutional Animal Care and Use Committee.

## Methods

### Preparation of RGD-LP-DTX

Liposomes were prepared by thin-film dispersion method. The steps were as follows: SPC, Chol, DSPE-PEG_2000_, DTX, and DSPE-PEG_2000_-RGD (molar ratio is 95: 20: 5: 5: 0.1) were dissolved in chloroform, and then placed in round bottom bottles to form uniform thin films (37 ℃, 90 rpm) via spinning and drying in a vacuum drying chamber at 37 ℃ for 2 h. Phosphate buffered saline (PBS) buffer (pH 7.4, 0.01 M) was added and hydrated in a rotary evaporator for 30 min (37 ℃, 90 rpm). Liposome was prepared by probe sonication (150 W) for 3 min [3]. PEG-LP-DTX was prepared using the same method adding SPC, Chol, DSPE-PEG_2000_, and DTX (molar ratio is 95: 20: 5: 5). Mal-LP-DTX was prepared using the same method adding SPC, Chol, DSPE-PEG_2000_, DTX, and DSPE-PEG_2000_-Mal (molar ratio is 95: 20: 5: 5: 1). Mal-RGD-LP-DTX was prepared using the same method adding SPC, Chol, DSPE-PEG_2000_, DTX, DSPE-PEG_2000_-RGD, and DSPE-PEG_2000_-Mal (molar ratio is 95: 20: 5: 5: 0.1: 1). The preparation of liposomes containing C6 or DiR done with fluorescent probes: C6 (drug-lipid molar is 1: 80) or DiR (drug-lipid molar ratio is 1: 30) was dissolved in chloroform solution of liposome materials, the other steps are the same as before [6]. The non-encapsulated drug from liposomes was removed by the method of dialysis.

### Preparation of RGD-Lf-LP-DTX

Lf-modified DTX-loaded liposomes (Lf-LP-DTX) were prepared by conjugating Lf to a distal Mal functional group on DSPE-PEG_2000_-Mal. Firstly, Lf was thiolated with an excess of Traut reagent (the molar ratio is Lf and Traut reagent was 0.1: 1). Thiolated Lf was added to Mal-LP-DTX or Mal-RGD-LP-DTX, and reacted for 4 h at room temperature to form a thioether bond with the Mal functional group at the N-terminus of the DSPE-PEG_2000_-Mal chain. The reacted liposome solution was eluted through a Sepharose CL-4B gel column at 0.01 mol L^−1^ PBS pH 7.4 to remove unbound Lf. Then Lf-LP-DTX and RGD-Lf-LP-DTX were prepared [3]. Lf grafting rate was determined by the BCA assay kit [16].

### Particle size, zeta potential, and morphology

The liposomes were diluted with ultrapure water to the appropriate concentration for measurement. The average particle size and zeta potential of the liposomes and their distribution was measured at 25 °C using a Nano Zetasizer dynamic light scattering (DLS) detector (Malvern, ZS90, UK). The range of particle size distribution was characterized by the polydispersity index (PDI), and the morphology of the liposomes was observed by transmission electron microscopy (TEM) (Hitachi, HT7700, Japan) [15].

### Characterization of EE, DLC and in vitro release

The EE and DLC of liposomes were determined by dialysis method. The dialyzate of 0.01 mol L^−1^ PBS at pH 7.4 was dialyzed (MWCO 14 kDa) for 6 h at 37 ℃ with a constant temperature shaker, and 20 μL of the liposome solution was injected into an HPLC system (Shimadzu, 20-AT, Japan). Hypersil BSD C_18_ reverse phase column (250 mm × 4.6 mm, 5 μm), and the mobile phase consisted of acetonitrile and ultrapure water (55/45, volume/volume). The target component was eluted at 25 °C by a mobile phase at a flow rate of 1.0 mL min^−1^ and monitored at 230 nm. The EE and DLC were calculated based on the dose, the weight of the carrier, and the measured DTX concentration [17].

$${\text{EE }}\left( \% \right) \, = {\text{Weight of }}\left( {{\text{total drug }} - {\text{ free drug}}} \right)/{\text{Weight of total drug }} \times { 1}00\%$$

$${\text{DLC }}\left( \% \right) \, = {\text{Weight of }}\left( {{\text{total drug }} - {\text{ free drug}}} \right)/{\text{Weight of total drug and carriers}} \times {1}00\%$$

In vitro release of the formulations was determined using the dialysis method. For example, 2 mL formulations were sealed into a dialysis bag (MWCO, 8000–12000 Da), and immersed into 30 mL pH 7.4 PBS containing 1 mol L^−1^ sodium salicylate at 37 °C while stirring at 100 rpm. The sample in the bag was removed at different time points for up to 72 h, than extracted with chloroform; DTX were determined by HPLC as described previously [18]. All experiments were carried out in triplicate.

### In vitro stability of liposomes

To evaluate the stability of liposomes in serum, each liposome has been incubated in 50% fetal bovine serum (FBS) at 37 °C for 24 h [19]. At pre-determined time points (1, 2, 4, 8, 12 and 24 h) liposome average diameter, Polydispersion index (PDI), and zeta potential were measured by Zetasizer nano (Malvern Instrument, Worcestershire, United Kingdom). 150 μL of the sample was pipetted and transferred to a 96-well plate, and the transmission at 750 nm was measured with a microplate reader (Thermo Scientific Varioskan Flash, USA).

A long-term stability investigation of liposomes stored at 4 ± 1 °C was performed by monitoring the liposome average diameter, PDI and zeta potential over 28 days [15].

### In vitro cellular uptake assay

For qualitative study of cellular uptake, U87 MG cells and hCMEC/D3 cells were seeded in 12-well plates (5 × 10^4^ cells/well). After growing overnight, the culture medium was replaced with 5 μg mL^−1^ C6 loaded PEG-LP, RGD-LP, Lf-LP, and RGD-Lf-LP for 4 h. Then cells were then washed with PBS, fixed with 4% (wt/vol) paraformaldehyde, and stained with Hoechst 33342; cellular uptake was imaged using fluorescence microscope (Zeiss, AXio lmager Z2, Germany). For quantitative analysis, cells were washed by PBS for three times, and the fluorescence intensity was detected by a flow cytometer [20] (BD, FACS AriaIII, USA).

### Mechanism of cellular uptake

U87 MG cells and hCMEC/D3 cells were seeded in 96-well plates at a density of 1 × 10^4^ per well, and cultured overnight. After adding the endocytosis inhibitor, amiloride (15 μg mL^−1^), chlorpromazine hydrochloride (10 μg mL^−1^), filipin (5 μg mL^−1^), colchicines (5 μg mL^−1^), RGD (200 μg mL^−1^), and Lf (1 mg mL^−1^), RGD + Lf (200 μg mL^−1^ RGD and 1 mg mL^−1^ Lf), were incubated at 37 °C for 30 min [15], then replaced with 5 μg·mL^−1^ RGD-Lf-LP-C6, and then finally incubated at 37 °C for 4 h. After that, they were washed with PBS 3 times for 5 min, 1% TritonX-100 was added for 40 min, and we then measured the fluorescence intensity at 466/505 nm with a fluorescent plate reader (Bio-Tek, USA).

### Penetration of tumor spheroids

U87 MG three-dimensional spheroids were built as previously reported [21]. U87 MG cells were seeded in 96-well plates with 5 × 10^3^ cells/well on sterile round coverslips pre-coated with 2% low melting point agarose. Six days later, the tumor spheroids were formed and C6 loaded PEG-LP, RGD-LP, Lf-LP, and Lf-RGD-LP were added to the culture dishes with tumor spheroids at the concentration of 20 μg·mL^−1^. After 4 h incubation, spheroids were washed with PBS for 3 times and fixed with 4% paraformaldehyde overnight. The fluorescent intensity of different depths of tumor spheroids was obtained through confocal laser scanning microscopy (CLSM) (Zeiss, LSM710, Germany).

### Cytotoxicity assay

MTT assay was used to measure U87 MG cells cytotoxicity in vitro. In brief, U87 MG cells were seeded into 96-well plates with 1 × 10^4^ cells/well and then incubated for 24 h. The culture medium was substituted with different liposomes at serial DTX concentrations for the next 24 h. After which, 20 μL of MTT solution (5 mg mL^−1^) was added to each well for further incubation. After further 4 h, the MTT solution was removed, and 150 μL of DMSO was added to each well to dissolve purple formazan crystals. The absorbance at 492 nm of each well was detected by a Microplate reader (Bio-Tek, USA). Untreated cells served as the control group [6]. Cell viability was calculated according to the following formula: cell viability (%) = A_sample_/A_control_ × 100%.

### Endothelial permeability test of hCMEC/D3 cells

Briefly, hCMEC/D3 cells, a density of 5 × 10^4^ cells/well, were cultured on a 12-transwell insert (pore size of 0.4 μm) coated with 1% type I collagen and grown in supplemented media. To verify cell barrier integrity, transendothelial electrical resistance (TEER) was determined using Millicell ERS-2 (Millipore, USA) prior to endothelial permeability experiments. The quality of the cell monolayer was tested by measuring the Lucifer yellow permeability as reported before [18]. The medium suspension of C6-loaded PEG-LP, RGD-LP, Lf-LP and RGD-Lf-LP was placed on the upper chamber and then incubated at 37 ℃ for 6 h. Endothelial permeability of C6-loaded PEG-LP, RGD-LP, Lf-LP and RGD-Lf-LP across cell monolayers was estimated by measuring the fluorescence intensity of the sample at different times (upper and lower chambers). To determine the apparent permeability values across blank Transwell inserts, experiments were performed in triplicate without seeding cells in the inserts.

### In vivo imaging in mice

The U87 MG orthotopic glioma model was established by injection of U87 MG cells (5 × 10^5^ cells/5 μL) into the right brain of BALB/c nude mice (2.0 mm laterally to the bregma, and 3.0 mm deep from the dura) using a stereotaxic apparatus (RWD, 68528 China). After 8 days postimplantation, 200 μL of DiR-loaded different liposomes (1 mg kg^−1^) were injected into the tail vein of BALB/c nude mice. After injection, fluorescence imaging of mice was performed at 4, 8, 12, and 24 h with the IVIS (PerkinElmer, Lumina III, USA). At 24 h postinjection, three mice in each group were sacrificed, and perfused with 0.9% physiological saline and 4% paraformaldehyde. The main organs, including the brains, hearts, livers, spleens, lungs, and kidneys were collected and analyzed with the IVIS [22].

### Tissue distribution

In vivo tissue distribution of liposomes was conducted on orthotopic U87 MG bearing nude mice as mentioned in the method of in vivo imaging. At 8 days after tumor inoculation, nude mice were divided into 4 groups randomly with 9 mice in each group, and was injected via the tail vein with PEG-LP-DTX, RGD-LP-DTX, LF-LP-DTX, RGD-LF-LP-DTX (400 μL, contains 0.5 mg mL^−1^ DTX). After 1, 4, 12, 24 h treatment, mice were sacrificed, and blood, heart, liver, spleen, lung, kidney, brain were taken out, respectively [23]. All tissues samples were washed by normal saline, then weighed and stored at −20 °C for measurement. The concentrations of DTX in plasma and homogenized tissues were determined as mentioned in the section of in vitro release.

### In vivo anti-tumor study

U87 MG cells were obtained and diluted to 5 × 10^6^ cells·mL^−1^ with DMEM. Subsequently, U87 MG cells (0.2 mL) were carefully injected subcutaneously into the right armpit of the BALB/c nude mice. When the tumor volume reached around 50–150 mm^3^, nude mice were randomly divided into 5 groups (n = 5) and treated with 0.9% Saline, PEG-LP-DTX, RGD-LP-DTX, Lf-LP-DTX, and RGD-Lf-LP-DTX every two day at a dose of 5 mg·kg^−1^ body weight. Tumor volumes and body weights were recorded every day after the first injection. The tumor volume was calculated as follows: tumor volume (mm^3^) = 0.5 × (length × width^2^). On the 12th day, all treated mice were sacrificed, and the tumors were excised, collected, and weighed. The liver and spleen were harvested, sliced, and stained with hematoxylin–eosin (HE) to observe drug toxicity. TUNEL assay was performed according to the manufacturer’s protocol [6].

In addition, we established orthotopic U87-MG glioma model in nude mice as reported [18]. At 6 day after tumor inoculation, nude mice were randomly divided into 5 groups with 8 mice in each group. The groups were as follows: Model, PEG-LP-DTX, RGD-LP-DTX, LF-LP-DTX, RGD-LF-LP-DTX. The mice in the treatment group were given via the tail vein with doses equivalent to DTX of 5 mg·kg ^−1^ every 3 days for 4 additional doses, the survival time and body weights of mice was recorded.

### Statistical analysis

The results were displayed as the mean ± SD, utilizing one-way ANOVA for assessing differences in mean values among more than two groups using Graphpad Prism 5 software *p* < 0.05 was considered statistically significant.

## Results and discussion

### Preparation and characterization of liposomes

**Fig. 1:**
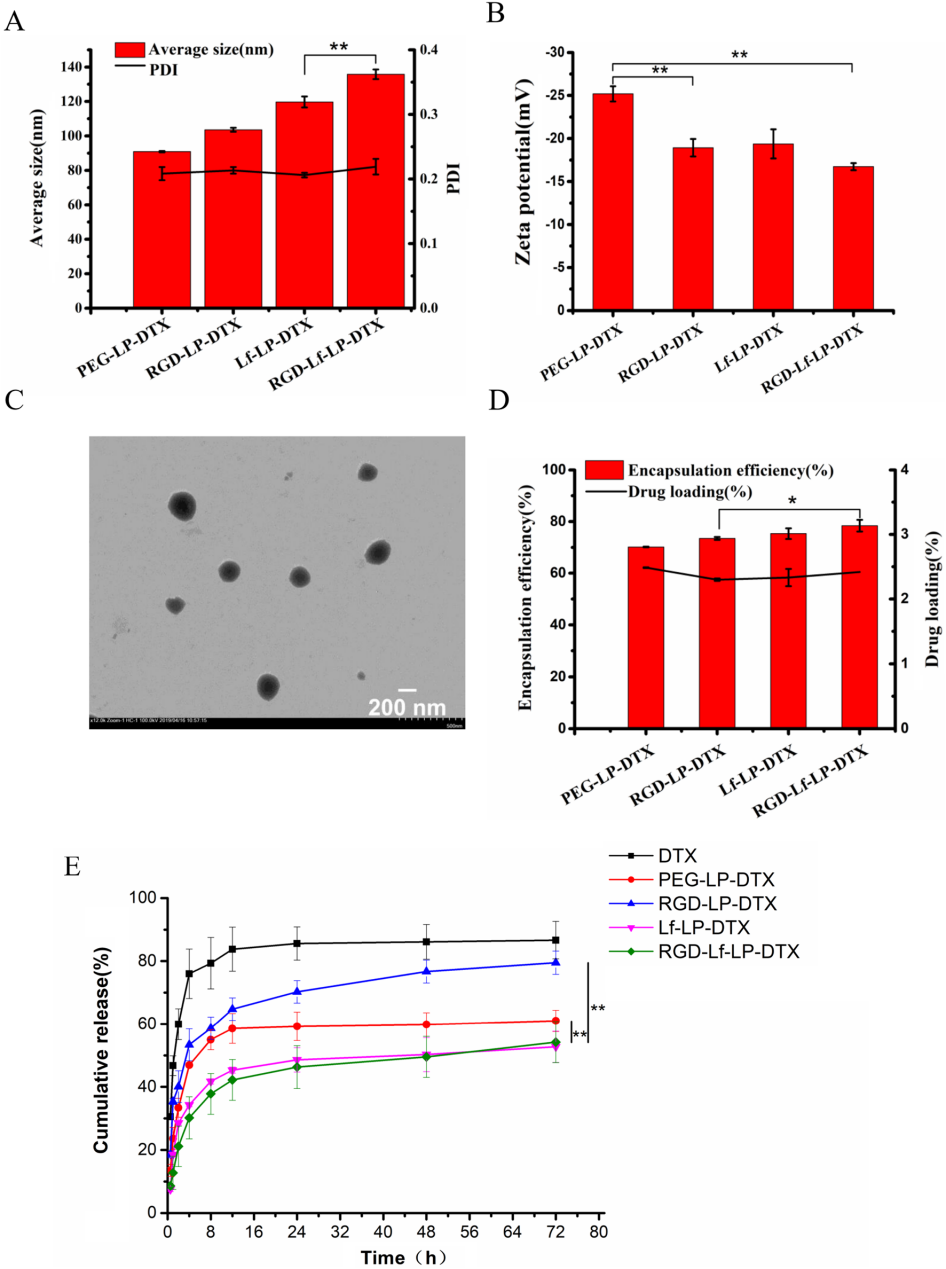
**A** Average size and PDI, **B** Zeta potential (**C**) TEM micrographs of RGD and Lf decorated, DTX-loaded nanostructured liposomes: RGD-Lf-LP-DTX (**D**) Encapsulation efficiency and drug loading of PEG-LP-DTX, RGD-LP-DTX, Lf-LP-DTX, RGD-Lf-LP- DTX. **E** The in vitro release profiles of formulations in PBS at 37 °C. Data represented the mean ± SD (*n* = 3), *p < 0.05, **p < 0.01

The particle size, zeta potential, polydispersity index, and EE and DLC of DTX-loaded liposomes are shown in Table 1. LF and RGD were used as ligands to successfully prepare RGD-Lf-LP-DTX; approximately 28 Lf molecules conjugated in per liposome were calculated based on the BCA protein assay kit, and 105 RGD molecules conjugated in per liposome were calculated according to the previously reported method [18, 24]. The particle size of PEG-LP-DTX, RGD-LP-DTX, Lf-LP-DTX, and RGD-Lf-LP-DTX were 90.9 ± 0.36 nm, 103.6 ± 1.15 nm, 119.7 ± 3.18 nm, and 135.8 ± 2.82 nm, respectively (Fig. 1A). The particle size of RGD-Lf-LP-DTX is approximately 136 nm, which was still in the scope for high uptake potential into glioma cells (below 200 nm) [1, 25]. The conjugation with RGD and Lf a modest increased the particle size.

**Table 1:** Characterization of DTX-loaded liposomes

Formulation	Particle size (nm)	Zeta potential (mV)	Polydispersity index	EE (%)	DLC (%)	Number of conjugated RGD in per liposome	Number of conjugated Lf in per liposome
PEG-LP-DTX	90.89 ± 0.36	−25.19 ± 0.89	0.208	71.17 ± 0.16	2.49 ± 0.01	–	–
RGD-LP-DTX	103.6 ± 1.15	−18.93 ± 1.01	0.213	73.49 ± 0.55	2.30 ± 0.02	105	–
Lf-LP-DTX	119.7 ± 3.18	−19.37 ± 1.70	0.206	75.34 ± 2.06	2.33 ± 0.13	–	28
RGD-Lf-LP-DTX	135.8 ± 2.82	−16.73 ± 0.42	0.219	78.42 ± 2.26	2.42 ± 0.01	105	28

Number of conjugated RGD in per liposome = n_RGD-DSPE-PEG2000_/(n_spc_*100,000)

The zeta potential of PEG-LP-DTX, RGD-LP-DTX, Lf-LP-DTX, and RGD-Lf-LP-DTX was determined to be −25.19 ± 0.89 mV, −18.93 ± 1.01 mV, −19.37 ± 1.70 mV, and −16.73 ± 0.42 mV, respectively (Fig. 1B). The zeta potential increased slightly after conjugated Lf and RGD. RGD had weak positive charge, small molecular weight, and less charge; Lf has negative charge, large molecular weight, and more charge [22, 26]. In addition, Lf with negative charge was neutralized by the thioylation reaction during the modification of liposomes, which may lead to the increase of the zeta potential in Lf modified liposomes. Consequently, the zeta potential of RGD-Lf-LP-DTX increased modestly.

TEM showed that liposomes were spherical vesicles, and additionally showed no aggregation phenomenon between particles (Fig. 1C). The apparent particle size observed by TEM was consistent with the previous result determined by DLS; namely, that the ligand modification had no significant effect on the morphology of the liposomes.

The EE values of all the formulations were over 70%. This result indicated that there was no significant difference in the EE of PEG-LP-DTX and RGD-Lf-LP-DTX, and demonstrated that the modification of Lf and RGD did not affect the drug encapsulation ability of the liposomes [3, 19]. The DLC of different liposomes varies from 2.2% to 2.5% (Fig. 1D).

The in vitro release profiles of DTX from the formulations are depicted in Fig. 1E. The results showed that DTX was rapidly released from the DTX solution with a release rate of 80% at 12 h, and PEG-LP-DTX showed a significantly sustained DTX release profile. Which might be due to the reason that the presence of additional PEGylated hydrophilic barrier retarded DTX release as reported [27]. However, RGD-LP-DTX exhibited slightly faster release properties than PEG-LP-DTX, which could be attributed to RGD enhancing the permeability of lipid bilayer membrane on the surface of liposomes. The release profile of DTX from LF-LP-DTX was slower than that of the PEG-LP-DTX, which may be explained by the electrostatic interactions of macromolecular Lf and DTX on the surface of liposomes that can hinder DTX release. RGD-Lf-LP-DTX and Lf-LP-DTX showed similar in vitro release curve, possibly due to the electrostatic interactions of macromolecular Lf and DTX play a larger role than RGD in the DTX release of LF and RGD dual modified liposomes. Moreover, the sustained-release behavior of DTX from RGD-Lf-LP-DTX may prolong the drug action time and enhance antitumor efficacy.

### In vitro stability of liposomes

**Fig. 2:**
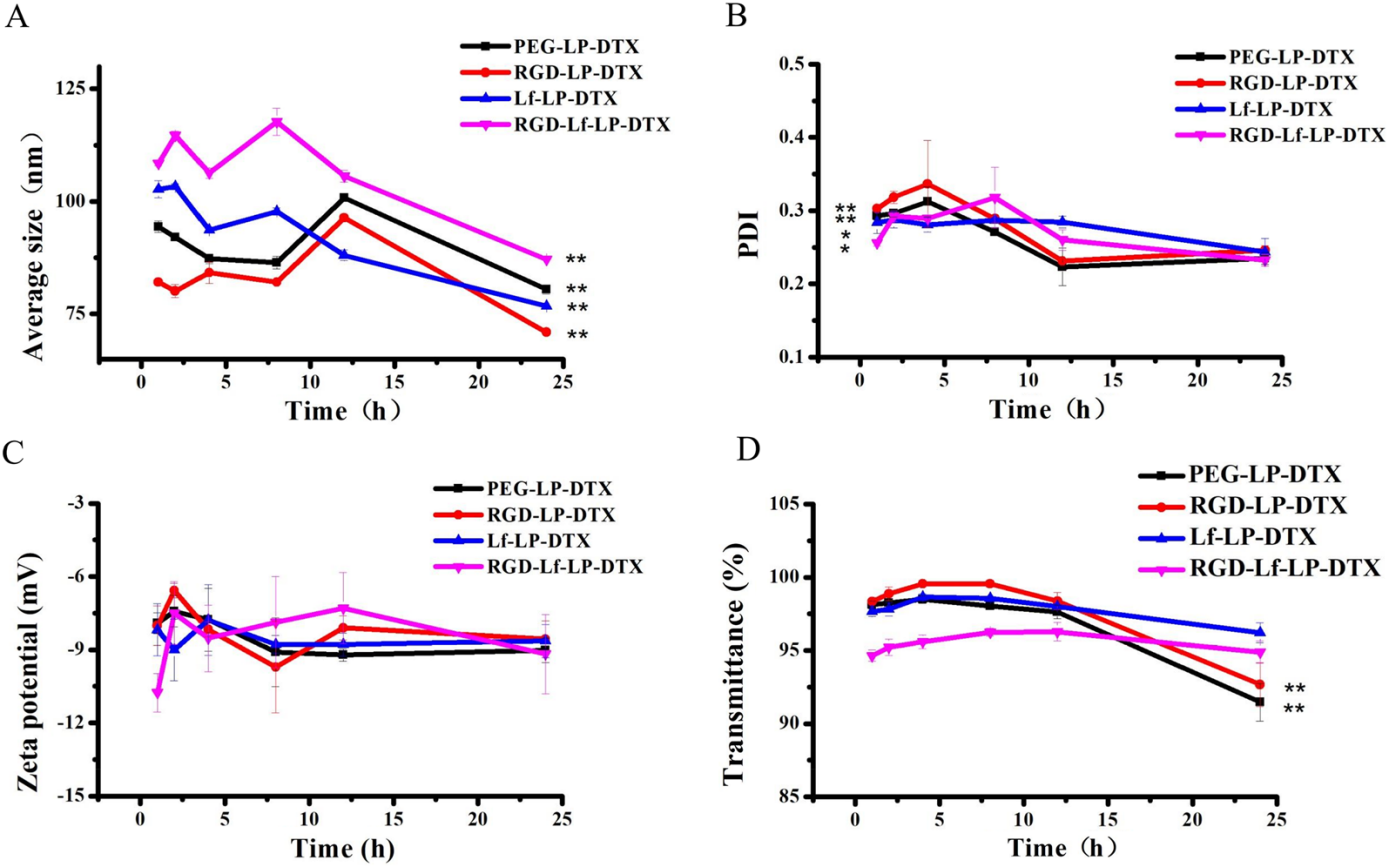
**A** The variation in average size, **B** PDI, **C** zeta potential, and **D** turbidity (represented by transmittance) of PEG-LP-DTX, RGD-LP-DTX, Lf-LP-DTX, and RGD-Lf-LP-DTX in 50% FBS. *p < 0.05, **p < 0.01 was significantly different with that of 1 h. Data is represented as mean ± SD (*n* = 3)

Average particle size, PDI, zeta potential, and transmittance variations as significant parameters were measured in our research to investigate the serum stability of liposomes. 50% FBS was used to simulate the in vivo serum situation. As seen in Fig. 2, the average particle size and PDI of PEG-LP-DTX, RGD-LP-DTX, Lf-LP-DTX, and RGD-Lf-LP-DTX decreased by less than 10% over 24 h, and the transmittance of PEG-LP-DTX and RGD-LP-DTX decreased by less than 10% over 24 h. Yet the transmittance of Lf-LP-DTX and RGD-Lf-LP-DTX has no significant change. While the zeta potential of RGD-Lf-LP-DTX changed significantly in the early stage, possibly because the residual free DSPE-PEG_2000_-RGD and LF were bound to some substances in 50% FBS [26], but returned to a stable state after 12 h, indicating that the liposome had good serum stability [28]. No aggregation was demonstrated in the presence of serum. The results showed that Lf and RGD modification did not affect the stability of liposomes.

**Fig. 3:**
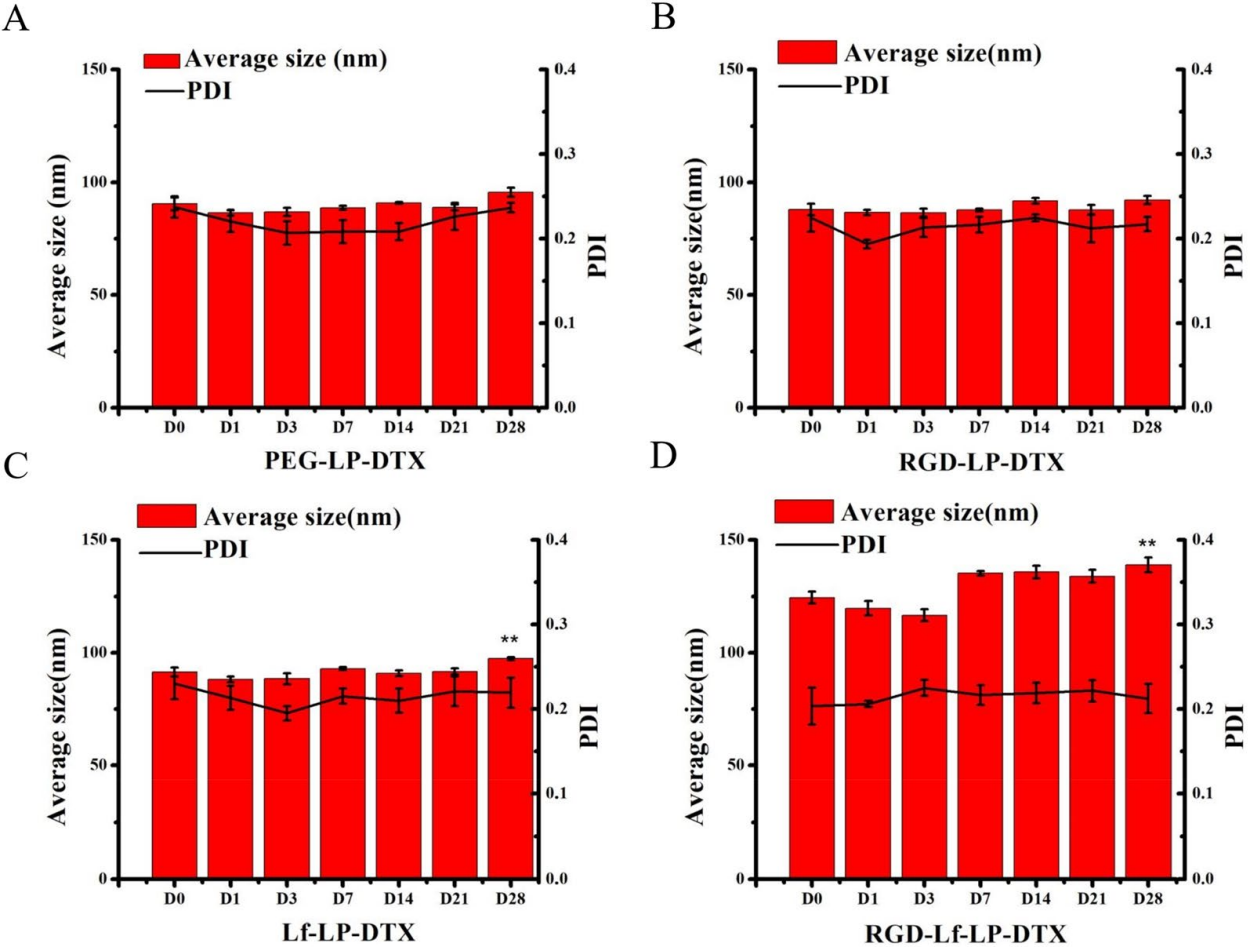
The stability of liposomes was evaluated by measuring average size and PDI for 28 days. **p < 0.01 was significantly different with that of 0 day. Data represented as mean ± SD (*n* = 3)

The stability of liposomes was investigated during a period of 28 days (Fig. 3). For PEG-LP-DTX and RGD-LP-DTX, the average particle size remained almost constant during the 28 day period. For Lf-LP-DTX and RGD-Lf-LP-DTX, the particle size increased by less than 10%. The PDI of all measured liposomes did not change significantly with time. So we think RGD-Lf-LP-DTX was stabilized at 4 °C during a period of 28 days.

### In vitro cellular uptake assay

**Fig. 4:**
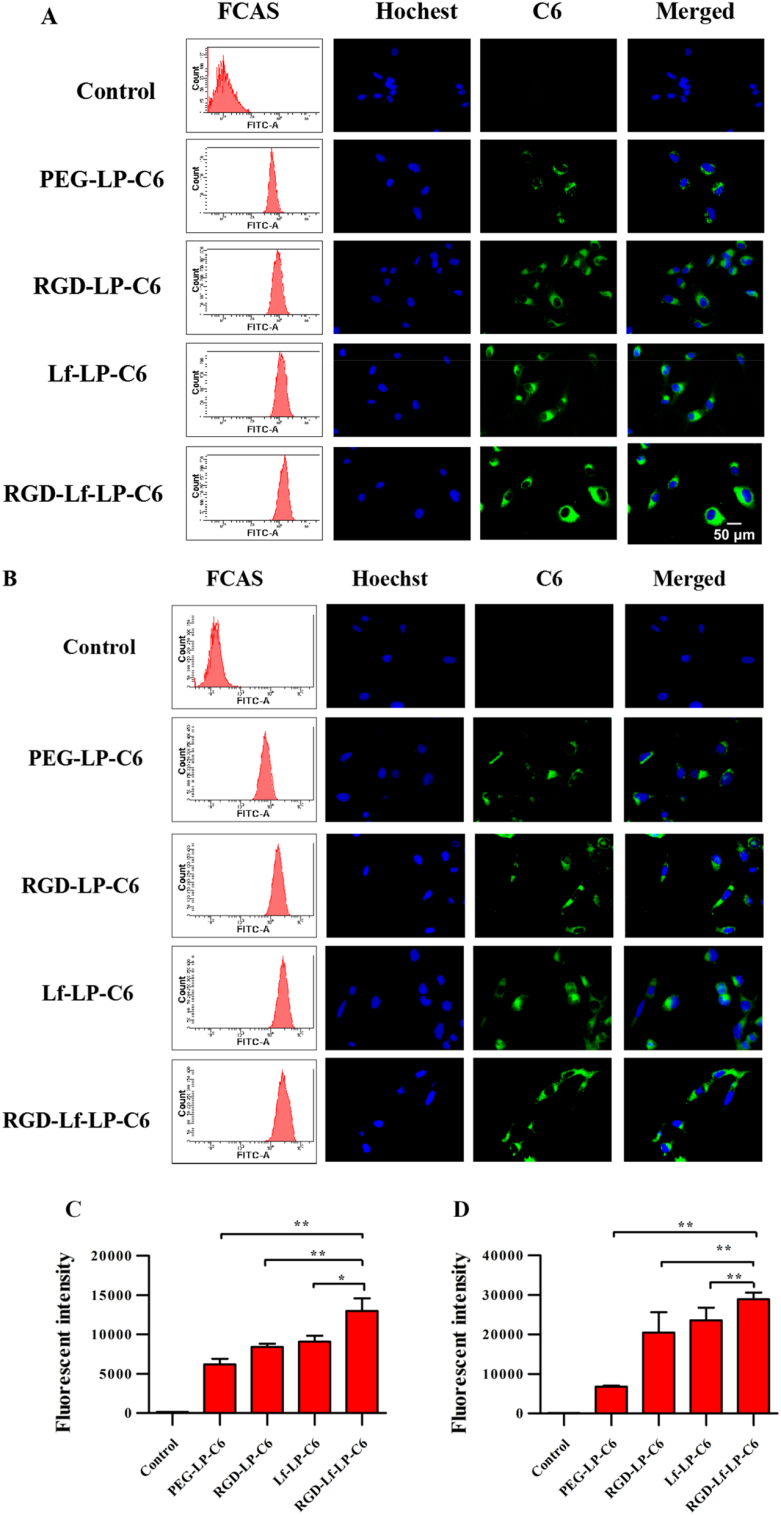
Cellular uptake of PEG-LP-C6, RGD-LP-C6, Lf-LP-C6, and RGD-Lf-LP-C6 in U87 MG cells (**A** and **C**) and hCMEC/D3 cells (**B** and **D**). Quantitative analysis was examined by flow cytometry (left panel of **A**–**D** *p < 0.05,**p < 0.01). Fluorescence microscopy images showed the Cellular uptake of PEG-LP-C6, RGD-LP-C6, Lf-LP-C6, and RGD-Lf-LP-C6 in U87 MG cells (right panel of **A**) and hCMEC/D3 cells (right panel of **B**). (Cell nuclei were stained blue with Hoechst 33342, and C6 loaded liposomes emitted green fluorescence)

Cellular uptake of C6-loaded liposomes in U87 MG and hCMEC/D3 cells was used to assess the targeting ability by fluorescence microscopy and flow cytometry. The fluorescence intensity of PEG-LP-C6, RGD-LP-C6, Lf-LP-C6, and RGD-Lf-LP-C6 increased in sequence, and the fluorescence intensity values of RGD-LP-C6, Lf-LP-C6, and RGD-Lf-LP-C6 in U87 MG cells were 1.43, 1.54, 2.10,-fold of PEG-LP-C6, respectively, and in hCMEC/D3 cells were 3.00, 3.46, and 4.24, -fold of PEG-LP-C6 (Fig. 4A and B). The qualitative and quantitative results were consistent in cellular uptake experiments. Furthermore, RGD-Lf-LP-C6 was found to have the highest fluorescence intensity in our cellular uptake experiments. Our results demonstrate that the RGD and Lf dual-modification enhanced the targeting ability of liposomes in both U87 MG cells and hCMEC/D3 cells. Higher cellular uptake could be attributed to Lf and RGD ligand-dependent endocytosis in U87 MG cells and hCMEC/D3 cells [3, 29].

### Mechanism of cellular uptake

**Fig. 5:**
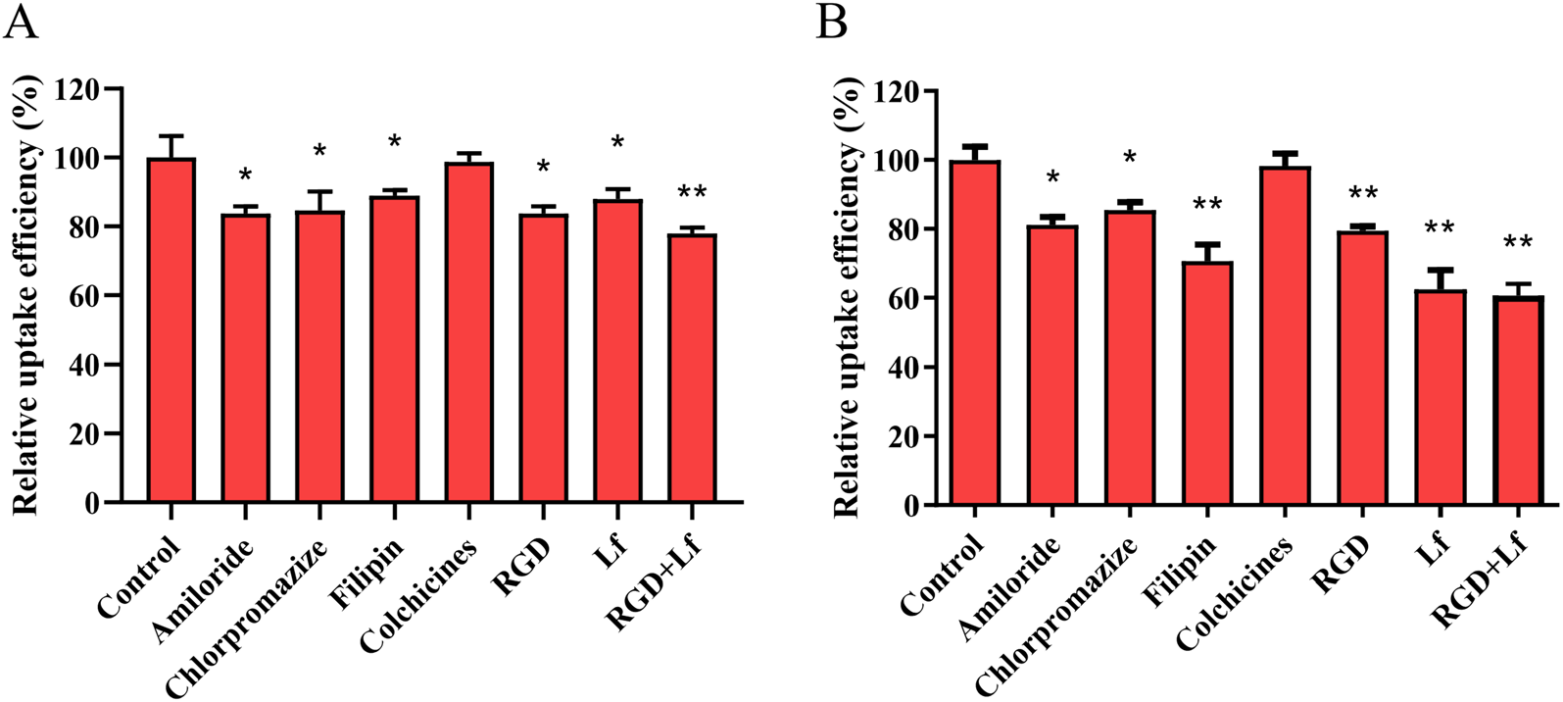
Cellular uptake of C6-loaded RGD-Lf-LP in the presence of different endocytosis inhibitors (amiloride, chlorpromazine hydrochloride, filipin, colchicines, RGD and Lf). Data represented the mean ± SD, *n* = 3, *p < 0.05, **p < 0.01 was significantly different with that of the non-inhibited control. **A **U87 MG cells, **B** hCMEC/D3 cells

The uptake efficiency of RGD-Lf-LP-C6 was decreased after pre-incubation of U87 MG cells and hCMEC/D3 cells with different endocytosis inhibitors. Among them, in U87 MG cells (Fig. 5A), the uptake of cellular with amiloride, chlorpromazine, filipin, RGD, Lf, and LF + RGD were 83.80%, 84.68%, 88.99%, 83.80%, 87.95% and 77.96% respectively. The relative uptake efficiency of these 6 groups was lower than that of the control group, and indicated that Na^+^/H^+^ exchanger, clathrin, and caveolin pathways were involved in cellular uptake. In hCMEC/D3 cells (Fig. 5B), the uptake was most decreased after incubation with amiloride, chlorpromazine, filipin, RGD, Lf, and LF + RGD, which were 81.17%, 85.40%, 70.63%, 79.50%,62.43%, and 60.67% respectively, it is suggested that Na^+^/H^+^ exchanger, clathrin, and caveolin pathways were involved in cellular uptake [26]. In addition, the results showed that free RGD and Lf competitively reduced the cellular uptake of RGD-Lf-LP, and RGD + Lf played most obvious inhibitory effect on reduced cellular uptake of RGD-Lf-LP. In particular, free RGD played a main inhibitory effect on cellular uptake of RGD-Lf-LP in U87 MG cells, yet free Lf played a main inhibitory effect on cellular uptake of RGD-Lf-LP in hCMEC/D3 cells. Which demonstrated that cellular uptake of RGD-Lf-LP in U87 MG and hCMEC/D3 cells involved integrin α_V_β_3_ receptor and LfR-mediated endocytosis [10, 31].

### Tumor spheroid penetration

**Fig. 6:**
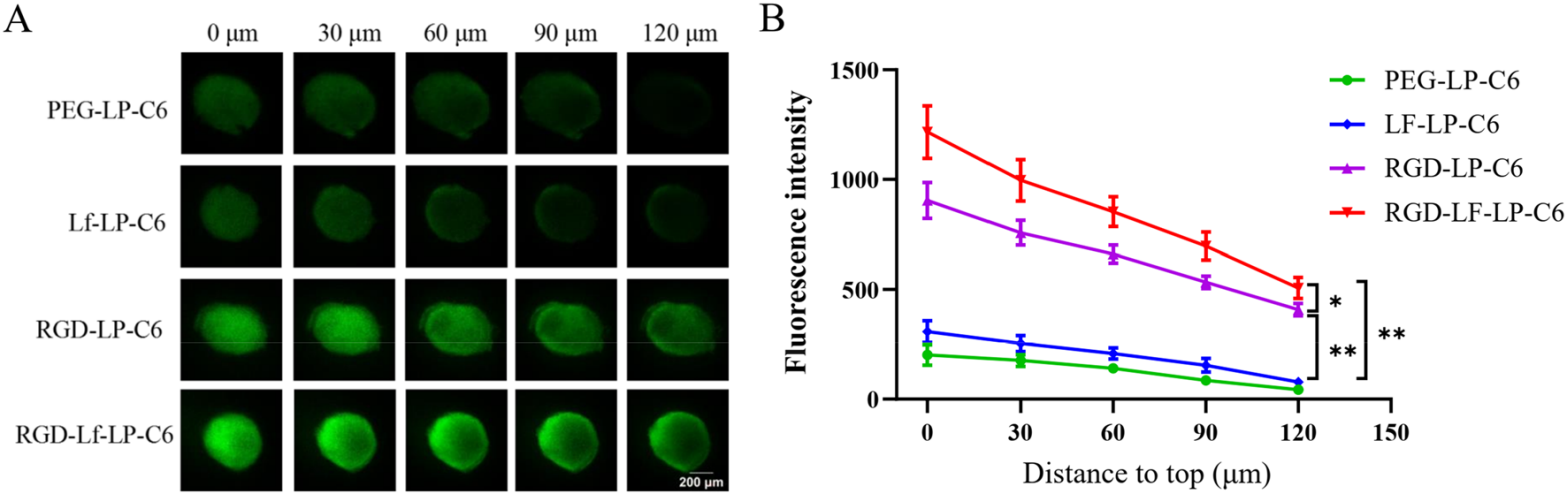
**A** Fluorescence images of U87MG glioma spheroids by the treatment of C6 loaded liposomes via confocal laser scanning microscopy (CLSM) from the top to middle layers (120 μm), bar representation 200 μm. **B** Semi-quantitative intensity of U87-MG tumor spheroids in each group (n = 3)

U87 MG tumor spheroids with different liposomes displayed different penetration effects. From the top to middle layers (120 μm), the fluorescence depth of different liposomes was PEG-LP-C6 < Lf-LP-C6 < RGD-LP-C6 < RGD-Lf-LP-C6 (Fig. 6). It is most significant that at the middle layers (120 μm) of the U87 MG tumor spheroids the fluorescence was only distributed in the margin of spheroids of PEG-LP-C6, Lf-LP-C6, and RGD-LP-C6, while the fluorescence was distributed in the core of U87 MG spheroids of RGD-Lf-LP-C6. Furthermore, the semi-quantitative intensity of U87 MG tumor spheroids in each group was calculated (Fig. 6B). Compared to Lf-LP-C6 group, the fluorescence signals of RGD-Lf-LP-C6 group and RGD-LP-C6 group markedly increased. The fluorescence intensity of RGD-Lf-LP-C6 was higher than that of RGD-LP-C6 (*p* < 0.05).

Interestingly, we found that the results of tumor spheroids penetration seem to be a little inconsistent with the results of in vitro cellular uptake. The tumor spheroid is formed by the compact growth of tumor cells, we speculated that Lf modified liposomes with large molecular space structure was difficult to enter the deep part of the tumor spheroids in a short time (4 h). Therefore, the fluorescence intensity of the tumor spheroids in Lf modified liposomes group was weak. In addition, RGD is a member of tumor-penetrating peptide homes, can be effectively used to deliver agents or drugs into tumor [32, 33]. So RGD can promote the penetration of RGD modified liposomes into the deep part of tumor spheroids, and RGD modification plays a dominating role on promoting the penetration of tumor spheroids. As a result, RGD and Lf dual modified liposomes can significantly increase tumor spheroid penetration.

### Cytotoxicity assay

**Fig. 7:**
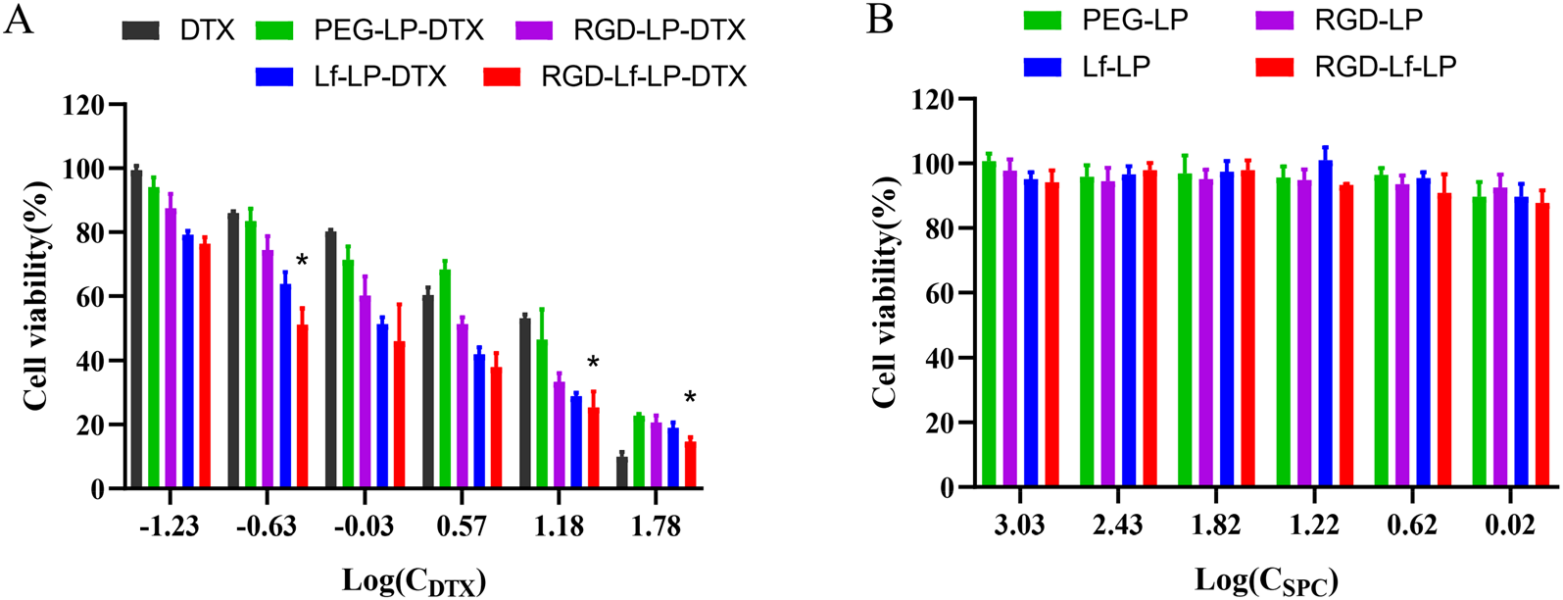
The cell viability of U87 MG cells cultured with free DTX, various DTX-loaded liposomes (**A**), and blank liposomes (**B**) at an equivalent DTX dose after 24 h, respectively. Data represented the mean ± SD (*n* = 3). *p < 0.05 compared with Lf-LP-DTX

We used an MTT assay to evaluate the cytotoxicity of different DTX-loaded liposomes on U87 MG cells. The half maximal inhibitory concentrations (IC_50_) were 18.53 μg mL^−1^, 11.08 μg mL^−1^, 2.49 μg mL^−1^, 1.30 μg mL^−1^, and 0.78 μg mL^−1^ for free DTX, PEG-LP-DTX, RGD-LP-DTX, Lf-LP-DTX, and RGD-Lf-LP-DTX, respectively (Fig. 7A). After 24 h treatment, no significant toxicity was found for blank unmodified and modified liposomes (Fig. 7B).

Our work demonstrated that all the DTX loaded liposomes showed higher cytotoxicity compared with free DTX, which might be attributed that liposomes or modified liposomes enhanced cell uptake and facilitated intracellular delivery of DTX subsequently [27]. This indicates that DTX-loaded liposomes inhibited glioma cell growth, and the inhibitory effects were further enhanced when using RGD and LF dual modified liposomes. The higher cytotoxicity of RGD-Lf-LP-DTX might be due to increased cellular uptake [30, 31].

### Endothelial permeability test of hCMEC/D3 cells

At 12–14 days inoculation, the TEER value of cell barrier was 68.5 ± 6.9 Ω·cm^2^ that was within the reference value of between 65 and 89 Ω·cm^2^ as reported [18], the cell monolayers were used for the further transport assay study. The permeability coefficient (P_app_) value of Lucifer yellow was 2.08 ± 0.26 × 10^–5^ cm/s, in agreement with reported values [18], indicating no adverse effect on cell monolayer integrity.

**Fig. 8:**
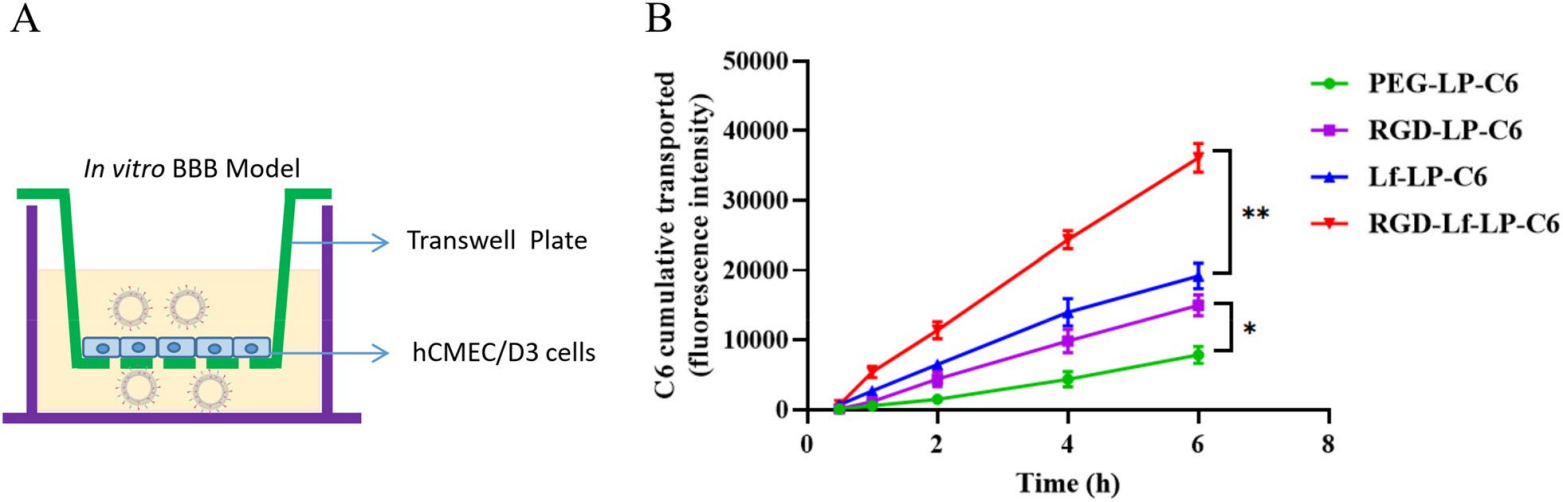
**A** Scheme of the Transwell model. **B** The C6 cumulative transported of C6 loaded liposomes through hCMEC/D3 cell monolayers at 0.5, 1, 2, 4 and 6 h

The C6 transport was evaluated as demonstrated before [31], the results were shown in Fig. 8. RGD-Lf-LP-C6 showed higher increases in C6 cumulative transported amount across in vitro BBB model than that of Lf-LP-C6 (*p* < 0.01) and RGD-LP-C6 (*p* < 0.01). The C6 transported amount of Lf-LP-C6 and RGD-LP-C6 were higher than that of PEG-LP-C6 (*p* < 0.05). The cumulative transported amount of Lf-LP-C6 is slightly higher than that of RGD-LP-C6 (no significant effect). The results were consistent with the cellular uptake by hCMEC/D3 cells. Due to the presence of integrin α_V_β_3_ receptor and LfR on the surface of hCMEC/D3 cells, the findings also inferred that RGD-Lf-LP-C6 increased transport of hCMEC/D3 cell monolayers by specifically recognizing integrin α_V_β_3_ receptor and LfR through receptor-mediated endocytosis pathways [10, 32] and Lf modification plays a dominating role on increasing the transport of hCMEC/D3 cell monolayers.

### In vivo imaging in mice

**Fig. 9:**
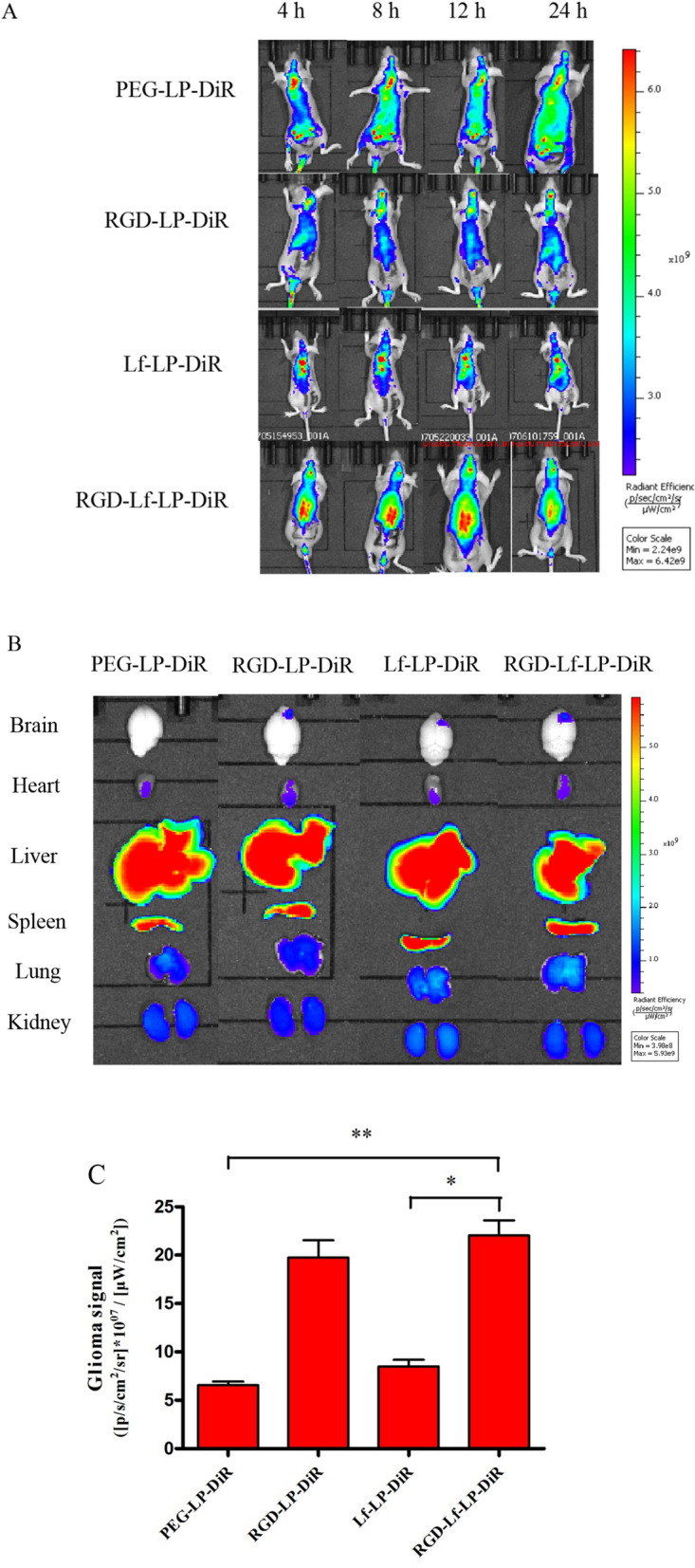
In vivo real-time imaging. **A** The U87 MG orthotopic tumor-bearing nude mice were given a tail vein injection of PEG-LP-DiR, RGD-LP-DiR, Lf-LP-DiR, and RGD-Lf-LP-DiR. All mice were scanned at 4, 8, 12, and 24 h. **B** The mice were sacrificed at 24 h, and the brains, hearts, livers, spleens, lungs, and kidneys were collected immediately. The fluorescence signal intensities in different organs were scanned. **C** Semi-quantitative intensity of glioma (injected site) tissues

The targeting ability of RGD-Lf-LP in orthotopic glioma was investigated via in vivo imaging in BALB/c nude mice. The real-time distribution of each group of liposomes in the glioma bearing nude mice was shown in Fig. 9A. The cerebral fluorescent signal of RGD-Lf-LP-DiR increased with time, and peaked at 12 h, when it was highest than at any other point in the post-injection period. The mice were sacrificed 24 h after tail vein injection, and the brains and main organs were collected for ex-vivo organ imaging (Fig. 9B). At the orthotopic glioma site, the fluorescence intensity of Lf-LP-DiR, RGD-LP-DiR, and RGD-Lf-LP-DiR are approximately 1.29-fold, 2.99-fold, and 3.35-fold higher than that of PEG-LP-DiR, respectively. As seen in Fig. 9C, RGD-Lf-LP-DiR displayed higher accumulation in tumors than the other groups. This was consistent with the results of tumor spheroid penetration. The results were mainly attributed to integrin α_V_β_3_ and LfR mediated endocytosis in U87 MG cells and hCMEC/D3 cells. However, Lf with large molecular weight and molecular space structure might have effect Lf modified liposomes penetrating into the deep tissue of the tumor. In addition, RGD with tumor-penetrating peptide properties can promote the penetration of agents into tumor [32, 33]. Therefore, RGD and Lf dual-modified liposomes showed most accumulation effects in brain tumor sites.

### Tissue distribution

**Fig. 10:**
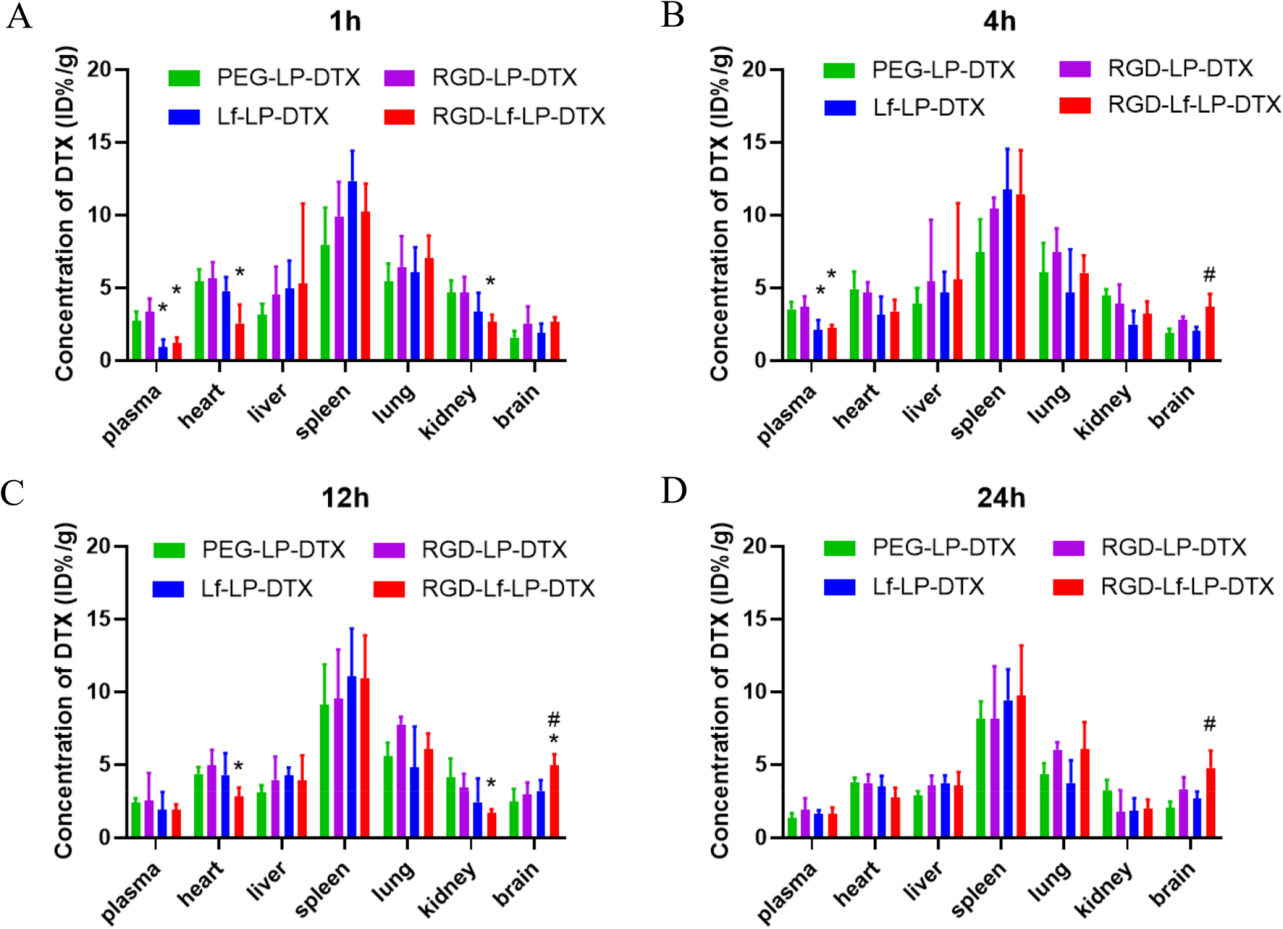
Tissue distribution of DTX after intravenous administration of DTX loaded liposomes, data represented the mean ± SD (n = 3). *p < 0.05 compared with RGD-LP-DTX, ^#^*p* < 0.05 compared with Lf-LP-DTX

As shown in Fig. 10, at 1 h and 4 h of administration, PEG-LP-DTX and RGD-LP-DTX group showed higher drug concentrations in blood than LF-LP-DTX group and RGD-LF-LP-DTX group, and the drug concentrations of RGD-LP-DTX was higher than that of PEG-LP-DTX.

The results attribute to RGD modification increasing in vitro release of liposomes, lead to higher drug concentration in plasma at 1 h and 4 h. The results were agreement with that of in vitro release. Compared with PEG-LP-DTX, dual-modified liposomes and single modified liposomes significantly increased the uptake by spleen. Furthermore, RGD-LF-LP-DTX showed higher drug concentrations in rat brain compared to the other treatment groups, especially at 12 h (*p* < 0.05), and RGD-LF-LP-DTX still showed higher drug concentrations in rat brain at 24 h (*p* < 0.05). The result indicated that RGD-LF-LP-DTX could significantly increase brain targeting after intravenous injection. In addition, RGD-LF-LP-DTX accumulated with less quantities of DTX in heart and kidney, which would reduce the toxic and side effects of DTX on the heart and kidney. The results of tissue distribution were basically consistent with that of in vivo imaging.

### In vivo anti-tumor efficacy

In vivo anti-tumor efficacy of liposomes was evaluated on subcutaneous and orthotopic glioma-bearing mice model. In subcutaneous model of glioma in nude mice, the anti-tumor efficiency of the dual targeted liposomes was assessed by measuring both the mean tumor volume in tumor-bearing mice and the inhibition rate of tumor weight. The tumor volume change curves (Additional file 1: Fig. S3A) showed that RGD-Lf-LP-DTX has better inhibitory effect on tumor growth than Lf-LP-DTX, RGD-LP-DTX, and PEG-LP-DTX. It is notable that RGD-Lf-LP-DTX achieved highlighted tumor-inhibition effect while tumor volume remained around 300 mm^3^. The tumor inhibition rate of PEG-LP-DTX was 38.84%, while the tumor inhibition rate of RGD-LP-DTX and Lf-LP-DTX was 64.31% and 70.42%, respectively (Additional file 1: Fig. S3C and D). RGD-Lf-LP-DTX showed stronger inhibition rate than Lf-LP-DTX, RGD-LP-DTX, and PEG-LP-DTX, which can be attributed to RGD and Lf dual-modified liposomes selectively targeting glioma and accumulating at the tumor site. In addition, the body weight of mice in every group displayed no evident difference during the experiment period (Additional file 1: Fig. S3B), which indicated that the formulations were without obvious toxicity. In addition, the in vivo anti-tumor effect was further evaluated by observing the apoptosis of tumor tissue. As shown in Additional file 1: Fig. S3E, tumor tissue apoptosis was measured by TUNEL assay. The mice group bearing glioma treated with normal saline was used as a negative control. The results displayed that RGD-Lf-LP-DTX induced stronger apoptosis than the PEG-LP-DTX, RGD-LP-DTX, and Lf-LP-DTX groups. Further, we investigated the histological examination of liver and spleen in Additional file 1: Fig. S4, the structure of hepatocytes in each group was normal without punctate or small patchy necrosis; the spleen tissue structure in each group was normal, the boundary between red pulp and white pulp was clear, and the lymphatic follicles were clear. Even if there are much formulations distribution in the liver and spleen, and the results of HE staining confirmed that there was no tissue toxicity in all formulations groups.

**Fig. 11:**
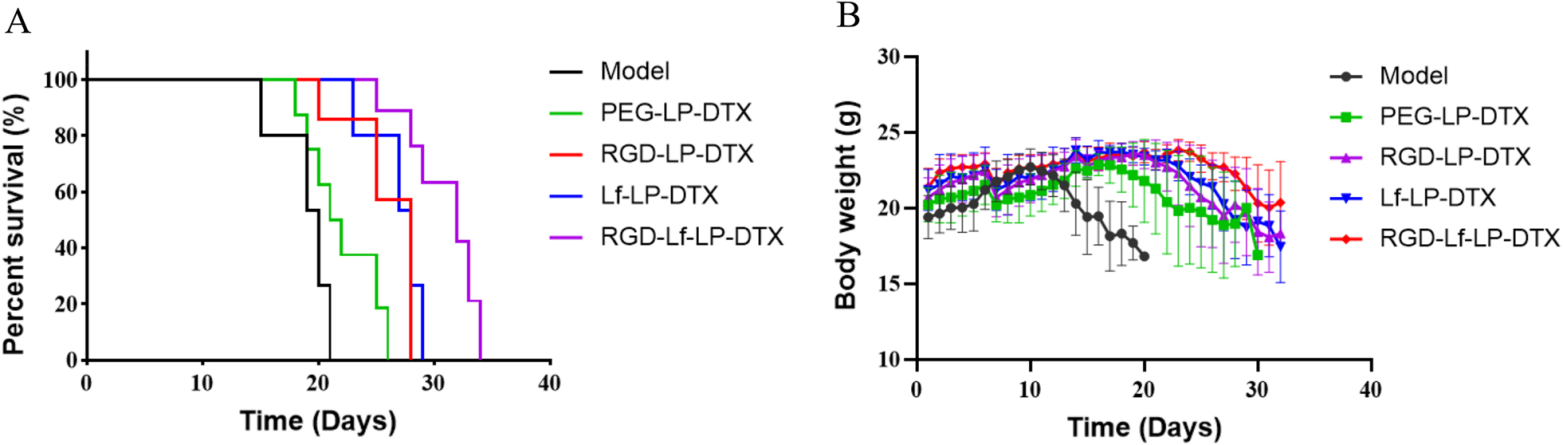
**A** Survival curves of U87-MG glioma bearing mice treated with different formulations (each dosing, 5 mg/kg DTX). RGD-Lf-LP-DTX group compared to RGD-LP-DTX and Lf-LP-DTX group, *p* < 0.05. RGD-LP-DTX or Lf-LP- DTX group compared to PEG-LP-DTX group, *p* < 0.05. **B** Changes in body weights of U87-MG glioma-bearing mice treated with different formulations

In orthotopic model of glioma in nude mice, as shown in Fig. 11A, median survival time of RGD-LF-LP-DTX (32 days) was significantly longer than that of model (20 days), PEG-LP-DTX (21.5 days), RGD-LP-DTX (28 days), and LF-LP-DTX (28 days) (*p* < 0.01). The result of Kaplan–Meier survival curve indicated that RGD and Lf dual modified liposomes loaded with DTX could significantly prolong the survival time of orthotopic glioma-bearing mice. However, from Fig. 11(A) and Additional file 1: Fig. S3(A), we also got the same result, the anti-tumor efficiency of Lf-LP-DTX is slightly better than that of RGD-LP-DTX (no significant difference). These results seem to be a little inconsistent with the results of tumor spheroid penetration and in vivo imaging. We speculate that the metabolism of LF modified liposomes with large molecular weight and spatial structure are relatively slow in the brain. As a result, LF modified liposomes have a longer effect on brain tumor sites, and DTX in liposomes continue to play an anti-glioma effect. We will continue to confirm this result in further research. Figure 11B showed that body weight of orthotopic glioma bearing mice no obvious differences in all treatment groups during experimental period. The result suggested that all the formulations were without obvious side effects. The anti-tumor efficacy on orthotopic glioma was consistent with the result of xenograft tumor model.

Given the overexpression of integrin α_v_β_3_ and LfR in glioma cells, RGD-Lf-LP-DTX enhances anti-tumor effects by increasing uptake of glioma cells through the integrin α_V_β_3_ receptor and LfR-mediated endocytosis [34–36]. Furthermore, as a member of tumor penetrating peptide homes, RGD can promote RGD-Lf-LP-DTX penetrating into tumor [20, 21]. Therefore, RGD and Lf dual modification increased the permeability and accumulation of liposomes in glioma, allowing more DTX to reach the glioma site, thus exerting the strongest anti-tumor effect [37].

## Conclusions

In this research, we developed integrin α_v_β_3_ and Lf receptor dual targeted, DTX loaded nanostructured liposomes for glioma therapy. As we have shown, RGD-Lf-LP-DTX is stable when nanosized, and has high encapsulation efficiency. RGD-Lf-LP-DTX also demonstrates higher cellular uptake, stronger tumor spheroid permeability, higher cellular cytotoxicity, can more effectively cross the BBB to reach brain tumors, and has obvious inhibition efficiency on xenograft glioma and could significantly prolong the survival time of orthotopic glioma-bearing mice. In summary, RGD and Lf dual modified liposomes loaded with DTX shows potential as a promising chemotherapeutic drug delivery system for glioma.

## Supplementary Information

**Additional file 1: Fig. S1.** The chemical structure of DSPE-PEG_2000_-RGD. **Fig. S2.** The ^1^H-NMR spectroscopy of DSPE-PEG_2000_-RGD. **Fig. S3.** Anti-tumor effects of DTX-loaded liposomes in vivo. (A) Growth curves of subcutaneous U87 MG tumor volume in nude mice given a tail vein injection with various DTX-loaded liposomes on the day 0, 2, 4, 6, 8,10 and 12; the total dose of DTX is 5 mg·kg^−1^. (B) Changes in body weight (C) Photographs of tumors at the end of treatments. (D) Tumor weight inhibition rate. (E) Tumor apoptosis cells were detected by TUNEL kit (nuclei and apoptosis cells are stained blue and red, respectively.) Data represented the mean ± SD (*n* = *5*, *p < 0.05, **p < 0.01). **Fig. S4.** HE staining of liver and spleen tissue, Magnification X200.
