# Supplementary material for: Combined integrin αvβ3 and lactoferrin receptor targeted docetaxel liposomes enhance the brain targeting effect and anti-glioma effect
Source: J Nanobiotechnology. 2021 Dec 23;19:446. doi: 10.1186/s12951-021-01180-0 (PMC8705194; doi:10.1186/s12951-021-01180-0)
Supplement: Supplementary file 1 — Additional file 1: Fig. S1. The chemical structure of DSPE-PEG2000-RGD. Fig. S2. The 1H-NMR spectroscopy of DSPE-PEG2000-RGD. Fig. S3. Anti-tumor effects of DTX-loaded liposomes in vivo. (A) Growth curves of subcutaneous U87 MG tumor volume in nude mice given a tail vein injection with various DTX-loaded liposomes on the day 0, 2, 4, 6, 8,10 and 12; the total dose of DTX is 5 mg·kg−1. (B) Changes in body weight (C) Photographs of tumors at the end of treatments. (D) Tumor weight inhibition rate. (E) Tumor apoptosis cells were detected by TUNEL kit (nuclei and apoptosis cells are stained blue and red, respectively.) Data represented the mean ± SD (n = 5, *p < 0.05, **p < 0.01). Fig. S4. HE staining of liver and spleen tissue, Magnification X200. [file 12951_2021_1180_MOESM1_ESM.docx]

**Supporting Information**

**Combined Integrin α_v_β_3_ and Lactoferrin receptor targeted docetaxel liposomes enhance the brain targeting effect and anti-glioma effect**

Na Qi^a,b#^, Shangqian Zhang^b#^, Xiantai Zhou^b#^, Wenjuan Duan^b^, Duan Gao ^b^, Jianfang Feng^c^, Aimin Li^a^*,

^a^ Cancer Center, Integrated Hospital of Traditional Chinese Medicine, Southern Medical University, Guangzhou, 510315, China;

^b^ Department of Pharmacy, Guilin Medical University, Guilin, 541004, China;

^c^ Department of Pharmacy, Guangxi University of Chinese Medicine, Nanning, 530299, China;

^#^These authors contribute equally to this work.

*Corresponding author.

E-mail addresses: liaimin2005@163.com (Aimin Li);

**Supporting Information Figure caption**

**Figure S1** The chemical structure of DSPE-PEG2_000_-RGD **S-2**

**Figure S2** The ^1^H-NMR spectroscopy of DSPE-PEG2_000_-RGD **S-2**

**Figure.S3** Anti-tumor effects of DTX-loaded liposomes in vivo. **S-3**

**Figure.S4** HE staining of liver and spleen tissue, Magnification X200. **S-4**


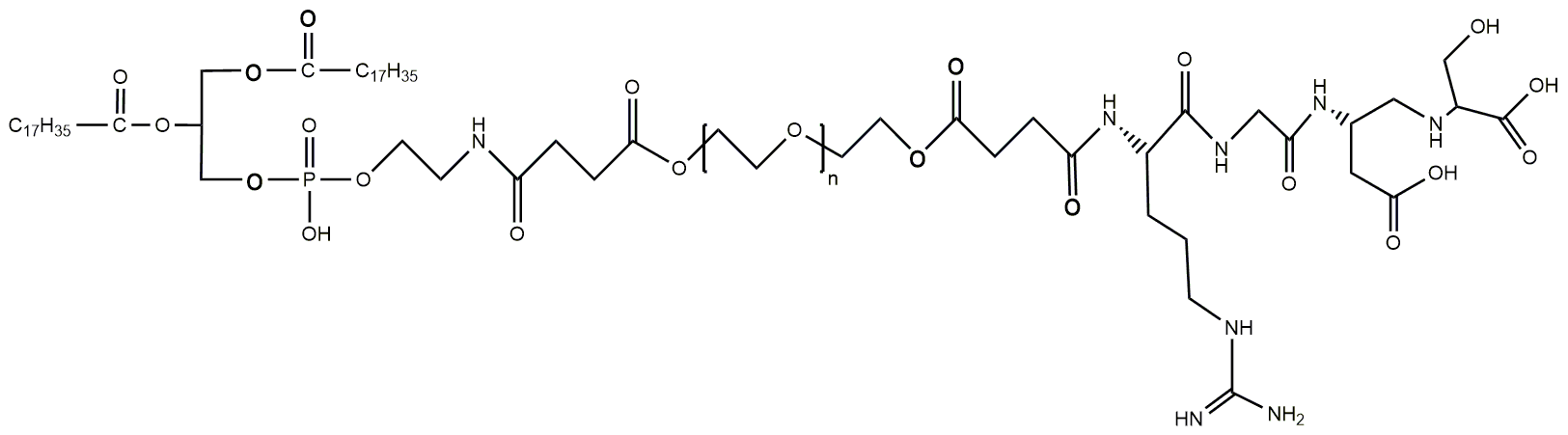


**Figure S1** The chemical structure of DSPE-PEG2_000_-RGD


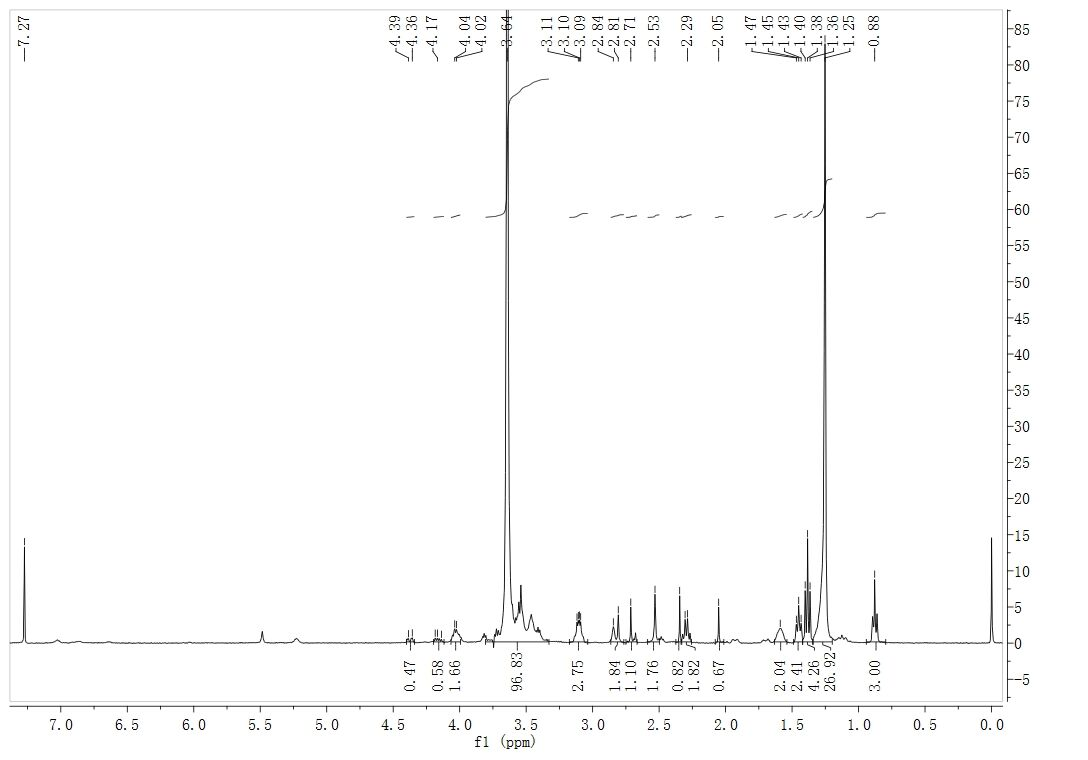


**Figure S2** The ^1^H-NMR spectroscopy of DSPE-PEG2_000_-RGD.


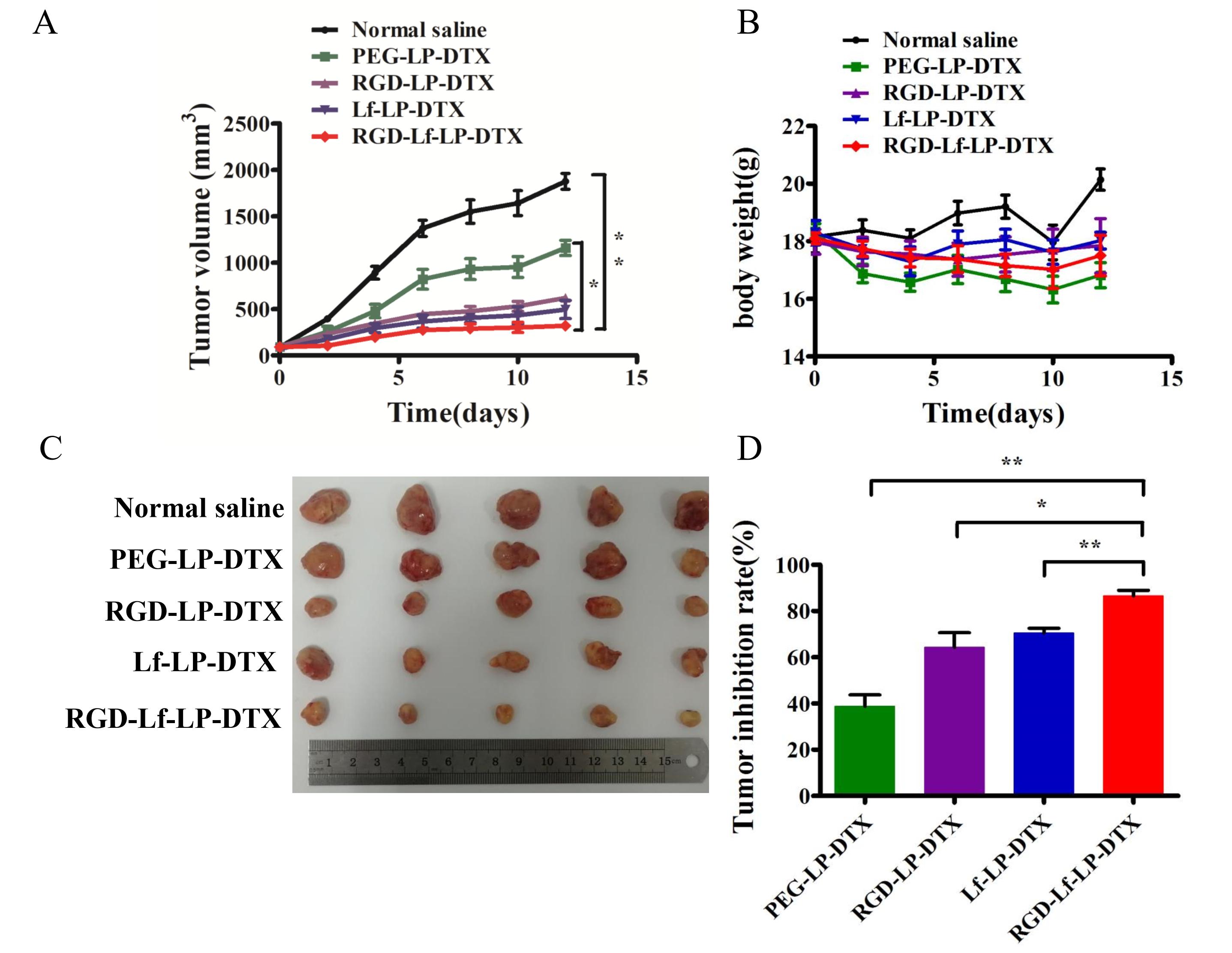


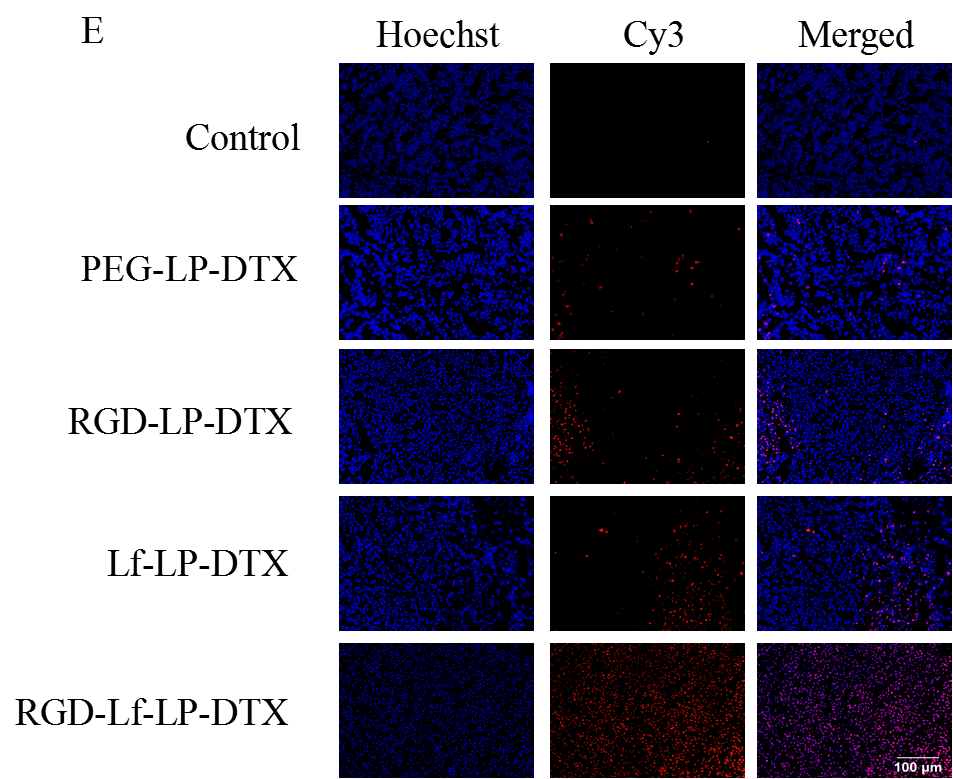


**Figure.S3** Anti-tumor effects of DTX-loaded liposomes in vivo. (A) Growth curves of subcutaneous U87 MG tumor volume in nude mice given a tail vein injection with various DTX-loaded liposomes on the day 0, 2, 4, 6, 8, 10 and 12; the total dose of DTX is 5 mg·kg^-1^. (B) Changes in body weight (C) Photographs of tumors at the end of treatments. (D) Tumor weight inhibition rate. (E) Tumor apoptosis cells were detected by TUNEL kit (nuclei and apoptosis cells are stained blue and red, respectively.) Data represented the mean ± SD (*n = 5*, **p* < 0.05, ***p* < 0.01).


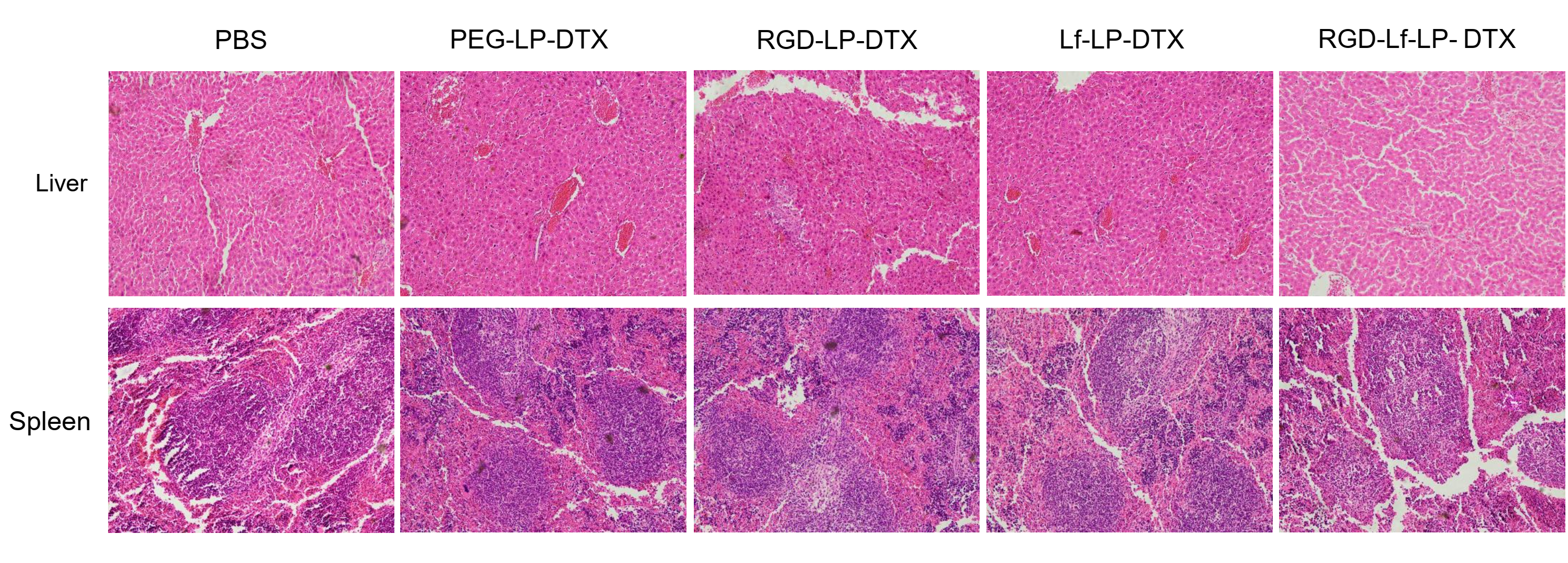


**Figure.S4** HE staining of liver and spleen tissue, Magnification X200

w
